# Inflammation and Cognition in Depression: A Narrative Review

**DOI:** 10.3390/jcm10245859

**Published:** 2021-12-14

**Authors:** Katarzyna Wachowska, Piotr Gałecki

**Affiliations:** Department of Adult Psychiatry, Medical University of Lodz, 91-229 Lodz, Poland; piotr.galecki@umed.lodz.pl

**Keywords:** depression, cognition, autobiographical memory, inflammation

## Abstract

The authors aim to present a narrative review of research on the inflammatory aetiology of depression. Depression is a psychiatric disorder, constituting the most common reason of disability due to a health condition. It has been estimated that at least one in six people suffer from depression at some point of their lives. The aetiology of depression, although researched extensively all around the world, still remains unclear. Authors discuss the possible role of inflammation in depression, the neurodevelopmental theory of depression as well as associations between cognition and depression. Possible associations between memory dysfunction among depressive patients and inflammatory markers are included. The associations between the immune system, depression and cognition are observed. Possible mediating factors between these areas include personality traits, hormonal imbalance and functioning of the brain areas. The question as to what mediating factors are involved is still open to research.

## 1. Introduction

Depression is a psychiatric disorder, constituting the most common reason of disability due to a health condition. More than 350 million people worldwide suffer from it. Life prevalence ranges from 14 to 18%. It has been estimated that at least one in six people suffer from depression at some point of their lives [1]. The disorder most often affects young people between 20 and 40 years of age. As early as 1990, depression was found to be the first cause of disability in the world and, according to WHO, by 2020 it was the second leading cause of disability resulting from a health condition in the world and the most common cause of death in every age group [2]. These findings turned out to be accurate, as shown in the GBD (Global Burden of Disease) 2015 study, in which depression was ranked as high as the third leading disease-causing disability resulting from a health condition in the world [3]. Ferrari et al., in a meta-analysis of world reports on the occurrence of depression, showed that the prevalence of depression is 4.7% [4]. Studies on the prevalence of mental diseases among the Polish population have shown that the DSM-IV criteria for major depression were met by 4.0% of women and 1.9% of men [5].

Depression is a risk factor of many complications, of which the most dangerous is suicide [6], and a reason of severe suffering among the people affected as well as their loved ones. The aetiology of depression, although researched extensively all around the world, still remains unclear. Depression was compared to the common cold disease in 2003 by Seligman, due to the speed of its spreading [7]. According to the contemporary scientific knowledge about depression, especially in the fields of immunology and neurochemistry, this comparison seems to be even more accurate. Today, psychiatrists tend to perceive depression as a chronic inflammation caused by stressors [8,9]. The associations between inflammatory markers and mood might also influence another important symptom of depression—cognitive disturbances.

The aim of the presented article was to review of research on links between inflammation, depression and cognitive deficits.

Two databases, Pubmed and Google Scholar, were searched for the defined terms “aetiology of depression”, “inflammatory theory of depression”, “interleukins and depression”, “depression and cognition” and “inflammation and cognition in depression”. The results were not limited in time frames as the goal was to describe the beginnings of the inflammatory theory of depression.

The article is divided into nine parts. Parts from two to eight describe main themes that were addressed in the literature, providing brief insight into it. Part nine is a summary.

## 2. Depression as a Disorder

Symptoms of depression can be observed in all areas of human functioning. For diagnostic purposes, the following groups of manifestations can be distinguished:

- Mood;
- Thinking and cognition;
- Motivation;
- Physical, also referred to as neurovegetative manifestations or symptoms, which take various forms [10]. It is indicated that people suffering from depression are more prone to developing somatic disease, and depression itself might be the first manifestation of infectious diseases, cancer and heart disease as well as a risk factor for a weaker therapeutic response and more hospitalizations [11,12,13].

The diagnostic criteria (according to DSM 5 and ICD 11) of depression are presented in Table 1 [14,15].

## 3. Pathogenesis of Depression—Three Main Groups of Factors

In the traditional approach, which is useful for academic purposes, the pathogenesis of depression includes the interaction of three factors: genetic predisposition, physical functioning of the body and exposure to stress factors. It is considered to be a result of the interaction between groups of biological and psychological factors, the influence of which cannot be considered separately. The group of biological factors includes genetic predisposition, changes of the levels of neurotransmitters and hormones and structural changes in the brain. Psychological factors include the content of internal conflicts in psychodynamic theories and changes in thinking (A. Beck’s cognitive triad and Seligman’s model of learned helplessness) [16]. Gałecki [17,18] emphasized the heterogeneity of the aetiology of depression and its multifactorial basis.

## 4. Inflammation and Depression

In the late twentieth century, several authors hypothesized the link between changes in the immune system function and depression. Several terms can be found in the literature:

- Macrophage theory of depression [19];
- The cytokine theory of depression, “the inflammatory response system model of depression” [20].

The current term used in the literature, reflecting the essence of the issue, is the inflammatory theory of depression [21,22]. Regardless of the name, this theory assumes the existence of interactions among the nervous, immune and neuroendocrine systems. The elements connecting and enabling these interactions are cytokines, large protein molecules that regulate processes at the cell level, including activation, proliferation, differentiation, movement, death [23,24] and communication, interaction and intercellular cooperation [24,25].

In addition to regulating the immune system, cytokines affect the metabolism of dopamine, noradrenaline and serotonin in the nuclei of the brain and increase cortisol secretion both through direct stimulation of the HPA axis and changes in the sensitivity of glucocorticosteroid receptors [26].

Shiepers et al. (2005) indicated that both the administration of cytokines for therapeutic purposes and the action of these substances during infection provoke in patients the picture of “*sickness behavior*”. This effect, in addition to typical flu-like symptoms (increased body temperature, headaches and muscle pains), includes symptoms characterizing depression: sleep disorders, decreased appetite, decreased motor activity, decreased libido, loss of interest, depressed mood, anhedonia and cognitive dysfunction [27]. Interestingly, symptoms disappear when the therapeutic administration of cytokines is discontinued [27].

Smith (1991) was one of the first scientists to associate the increased secretion of cytokines by macrophages with depression. He indicated that the cytokines administered to the subjects induced symptoms of depression, and interleukin-1 may provoke hormonal disorders associated with depression. He also pointed out that such an association could explain the important links between diseases underlying inflammation and macrophage activation and depression. He gave such examples as coronary artery disease and rheumatoid arthritis. He also emphasized that estrogens have the ability to activate macrophages, which may be responsible for the much higher incidence of depression among women [19].

At almost the same time—in the early nineties of the 20th century—Maes and his co-workers [28] showed that in people with a severe depressive episode the level of IL-6 is higher than that of healthy people and those with moderate severity of symptoms. They also demonstrated a correlation of this level with haptoglobin and transferrin and cortisol in the test inhibition with dexomethasone. This indicates the existence of a relationship in the level of IL-6, the severity of depressive symptoms and the hyperactivity of the HPA axis [28]. Maes [29] also showed that there is a correlation between IL-1beta levels and cortisol levels in the dexomethasone inhibition test. These results suggest that the observed dysregulation of HPA axis functioning is caused by the action of cytokines [29,30]. These interactions affect neuronal transmission and the functioning of the HPA axis [27], which results, among many other consequences, in a disruption of the kynurenine pathway [22,31]. The kynurenine hypothesis of the aetiology of depression indicates that inflammatory factors cause the excessive activation of indoleamine-2,3-dioxygenase. It is an enzyme present in the microglia, astrocytes and neurons, which catabolizes tryptophan into kynurenine, a substance toxic to the brain [4,5,6,7,8,9,10,11,12,13,14,15,16,17,18,19,20,21,22]. In addition to this toxic effect, the consequence of this process is the reduction in tryptophan levels needed for the production of serotonin.

## 5. Neurodevelopmental Theory of Depression

The aetiology of depression is complex and multifactorial, including dysfunctions in the serotonergic system, changes in the functioning of the hypothalamic–pituitary–adrenal axis and issues in the field of immunology [22,31]. One of the approaches that aims to integrate the existing ones is the neurodevelopmental theory [4,21]. According to its assumptions, a number of mechanisms in the course of individual development at the early stages of life determine susceptibility to depression in adulthood. The authors emphasized that as early as in the prenatal period, the influence of factors such as infections, maternal exposure to stressors and her ability to cope with them may underlie epigenetic mechanisms that affect gene expression without modifying the genetic code. In subsequent stages of individual development—early childhood and adolescence—the formation of personality and the development of brain areas are observed. The authors proposed that infections, stressors, and personal experiences interacting in this period have a comprehensive impact on the developed mechanisms of coping with stress. If the process develops a tendency to react with anxiety in many life situations, it causes hyperactivity of the HPA axis and chronic inflammation (neuroinflammation) and then a reduction in the size of the areas responsible for the regulation of emotions (hippocampus, more broadly the limbic system) and behavior regulation (frontal lobes). As a result, the following are observed: anhedonia, so-called “cold” deficits, such as attention, memory, executive functions, deficits in social functioning and “hot” deficits, such as emotional prejudices. As a result, we observe clinical symptoms of depression [4,5,6,7,8,9,10,11,12,13,14,15,16,17,18,19,20,21,22].

## 6. Interleukins and Depression—A Review of Prominent Examples

There is a lot of research on the role of particular interleukins in the development of depression. For this work’s purposes, we decided to present the best explored ones: IL-1 and IL-6.

Interleukin 1 is a family of cytokines that are characterized by multidirectional, mainly pro-inflammatory effects. They activate the endothelium, increase the production of acute phase proteins, increase body temperature, increase the production of antibodies by B lymphocytes and participate in many other processes [32].

People suffering from depression [33,34,35] as well as those diagnosed with dysthymia [36] have an increased concentration of IL-1beta compared to healthy ones [33,34,35], and the concentration of IL-1beta correlates with the severity of depression symptoms [33]. The level of IL-1beta decreases with the use of pharmacotherapy of depression with SSRI drugs [35,37], as well as during the augmentation of pharmacotherapy with electroacupuncture [34] and curcumin [38], and the decrease in the level of IL-1 beta correlates with lower severity of depressive symptoms [39], but this effect is not confirmed in all studies [36,40,41]. It was also shown that the enhancement of SSRI pharmacotherapy with acupuncture reduced the level of IL-1beta compared to the group using only pharmacotherapy [42,43]. Clinical improvement without a simultaneous reduction in IL-1 beta and IL-6 levels was achieved with fluoxetine treatment with or without omega-3 fatty acids [40]. The level of IL-1beta may be a predictor of individual response to antidepressant treatment—pharmacotherapy [44]—as well as the effectiveness of pharmacotherapy support with physical exercise [45].

Stress and the individual response to it increase the level of IL-1beta [46]. This increase may be modulated by dispositional optimism as a way of coping with a stressful situation. It is also a predictor of the development of depressive disorders in the future [47]. It has been shown that in veterans diagnosed with PTSD, the level of IL-1beta is elevated and correlates with the duration of the disease symptoms [48]. A summary of examples of studies on IL-1 levels with different kinds of co-factors are presented in Table 2.

Interleukin 6 is a pleiotropic cytokine that is a major modulator of the inflammatory response and hepatic production of acute phase proteins. It is also responsible for stimulating the hypothalamic–pituitary–adrenal axis in response to stress. Its concentration, both in blood and saliva, positively correlates with widely understood psychosocial risk factors such as cynicism, a tendency to experience feelings of powerlessness and anger and a sense of physical exhaustion [49], as well as depression and distress [50]. In contrast, it correlates negatively with psychosocial resources including coping skills and high self-esteem [49] and disposable optimism [51]. Elevated levels of IL-6 have been demonstrated in people under stress in laboratory conditions [46], those diagnosed with depression [35,52,53,54], as well as in people diagnosed with seasonal affective disorders [55] and among elderly people suffering from depression [56] as well as among women in puerperium [57]. Chinese studies did not confirm the increased concentration of IL-6 in the blood of people with depression [3]. However, the meta-analysis of longitudinal studies showed that the increased level of IL-6 may precede the development of depression [58] and is elevated in people with clinical symptoms, although not always unequivocally [59]. Somatic symptoms of both depression and anxiety especially correlate with high levels of inflammatory markers, including IL-6 [60]. The administration of endotoxin to healthy volunteers caused an increase in the level of IL-6 in the cerebrospinal fluid, which positively correlated with the severity of depressive symptoms consistent with a more severe depression of mood [61]. In a study by Basterzi et al. (2005), the level of IL-6 did not differ between depressed and healthy subjects; however, after 6 weeks of treatment with SSRI, there was a statistically significant decrease in the level of IL-6 in the group of patients [62], and when the symptoms of depression decreased during treatment, a decrease in the level of IL-6 is observed [63]. The meta-analysis of a world reports indicates that there is a relationship between treatment with SSRIs and a decrease in IL-6 levels [37]; such results were obtained for sertraline [64,65], as well as sertraline and citalopram [35], although these results are not unequivocal and repeatable in all studies [66]. Different results were obtained in a study on the effects of treatment with desvenlafaxine [67] and fluoxetine [40]. Surprisingly, in a study on the use of ketamine in the treatment of patients with treatment-resistant depression, the opposite result was obtained—an increase in IL-6 concentration, which did not correlate with the improvement of the condition of patients [68]. It was also shown that the enhancement of SSRI treatment with acupuncture reduced the level of IL-6 compared to the group using only pharmacotherapy [42,43] as well as support for typical pharmacotherapy of depression with celecoxib [69], acupuncture [42] and yoga and meditation [70], which were shown to reduce the concentration of IL-6. A similar effect was not observed for zinc supplementation [71], exercise [39], vitamin supplementation [72] or omega-3 fatty acids [40] used to support standard pharmacological treatment of depression. It has also been shown that the level of IL-6 in peripheral blood may be a predictor of response to antidepressant therapy. Such an association has been demonstrated for amitriptyline [73], exercise to support pharmacotherapy [45] and sleep deprivation therapy [74] but not for exercise used to enhance cognitive behavioral therapy [75]. Perhaps genetic variants of the genes encoding IL-6 play a role in the response to some drugs used in depression. Such a relationship was sought for duloxetine [76] and in the intensification of depression symptoms induced by interferon alpha therapy [77] as well as in patients treated with cognitive behavioral therapy [78]. A German study comparing different variants of cognitive-behavioral therapy and a group that did not undergo such a therapy, however, did not confirm such a relationship [75]. Similarly, light therapy of seasonal disorders to alleviate symptoms of depression did not reduce IL-6 levels [55]. In a study of patients with breast cancer, the level of IL-6 correlated with the severity of depressive symptoms [79]. In a study on the effects of cognitive behavioral therapy, pharmacotherapy, or a combination of both, IL-6 increased, rather than decreased, with improvement in patients’ condition [80]. In a study involving a group of oncological patients with depression symptoms, it was shown that psychological intervention in the form of group therapy decreased both the symptoms of depression and the level of IL-6 [81]. A summary of examples of studies on IL-6 levels with different kinds of co-factors is provided in Table 3.

An extensive meta-analysis of world reports largely confirms the positive correlation of the levels of some of the inflammatory markers, such as IL-1, IL-6, CRP and TNF-alpha with depression [82,83]. The presented interactions among these molecules and depression are numerous. The above considerations give backgrounds for perceiving cytokines as possible pathophysiological markers of depression. 

However, questions remain:

- Where does the inflammatory process come from? [84]
- Does correlation mean the existence of a cause-and-effect relationship? [58,85]

## 7. Cognition in Depression

In the group of patients suffering from recurrent depressive disorders, numerous cognitive disorders were observed. They are believed to be a very important component of the disorder, and were therefore included in the diagnostic criteria in DSM 5: “diminished ability to think or concentrate, or indecisiveness, nearly every day (either by subjective account or as observed by others)” as well as ICD 11: “difficulty concentrating” [14,15,86,87].

Rock et al. (2014), in a systematic review and meta-analysis, revealed that disturbances in cognitive functioning were observed among currently depressed patients as well as remitted patients. The affected functions included executive functioning, memory and attention [88,89]. Research indicates that cognitive deterioration is already present in the patients during the first episode of depression. The disorders concern the speed of information processing, working memory [86,87,88,89,90], executive functioning (action planning, inhibition of action, change of the way of action), verbal fluency, the ability to learn new information, delayed and direct auditory memory [89] as well as psychomotor slowing down and hand–eye coordination [21,22,91]. Importantly, cognitive impairment is observed during the remission of depression, when symptoms of low mood are no longer present [88]. Another systematic review [92] indicated that depression was associated with impairment in each kind of executive function taken into account, which included inhibition, mental flexibility, updating of information in working memory, planning, verbal fluency processing speed and vocabulary [92]. Świtalska et al. (2013), in a study involving a group of patients with bipolar disorder during depression, showed that the results in neuropsychological tests were not related to the intensity of depressive symptoms but to the number of hospitalizations, age of the onset and its duration [93]. Krogh (2014) presented association between level of inflammatory marker hsCRP and lower psychomotor speed among depressed patients [49]. Wesnes et al. (2016) described a study linking the level of memory processes functioning with the 5-HT1A receptor genotype. They showed that in the group of patients suffering from depression, a specific genotype of receptor polymorphism is associated with better maintenance and retrieval of information from episodic and working memory [94].

A meta-analysis of global reports analysing the cognitive functioning of patients with depression showed that the intensification of depression symptoms negatively affects cognitive functioning in the area of episodic memory, executive functions and data processing speed [95].

Changes in cognitive functioning during the treatment of depression among the elderly may be associated with the response to pharmacotherapy with citalopram [96] and may also be a risk factor for a worse response to fluoxetine treatment [97]. A summary of examples of research papers and meta-analyses on cognitive functioning among patients suffering from depression are presented in Table 4.

## 8. Conclusions

It has been observed that the impairment of both the immune system and cognition is present in the course of depressive disorder. The key observations presented in this article publications include:

- Increase of inflammatory markers in depressed patients is well established in many studies in the field of neuroimmunology.
- Research focused on cognitive domains suggests that at least some areas of cognitive functioning might be deteriorated in that group of patients.

Nevertheless, the cause of the observed abnormalities remains unclear. Furthermore, the possibility of whether they can be a potential target of treatment strategies is still undergoing discussions. Important questions that need to be answered include determining whether those abnormalities are cause–effect observation or whether there might be any co-factors.

**Figure 1:**
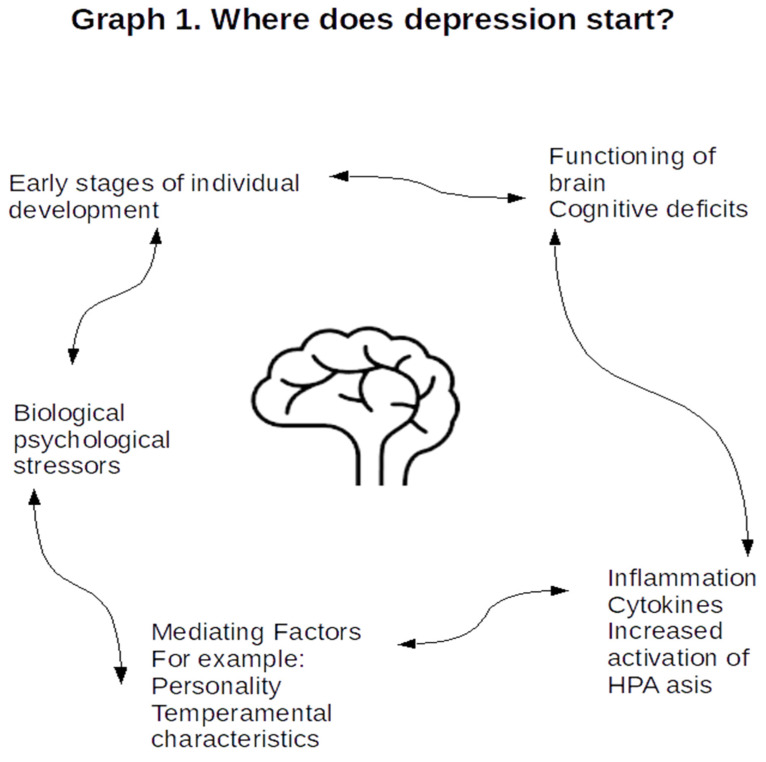
Possible multifactorial interactions, worth further investigation.

There are many possible co-factors of the observed relations, including personality traits, temperamental factors, structural abnormalities of the brain, hormonal imbalance and genetic predispositions. Some of them have been addressed in studies, for example, structural abnormalities of the brain. It is worth noting that the meta-analysis of the data on the size of the hippocampus in patients with depression showed that it is reduced in both hemispheres of the brain and may be related to the number of episodes of depression [98]. The hippocampus is one of the key centers related to memory [98]. It is worth emphasizing that it is an area that is particularly susceptible to the harmful effects of cortisol—a hormone whose high and prolonged level is associated with depression [98]. This structure is particularly susceptible to the harmful effects of glucocorticosteroids, and as a result of subsequent episodes of depression, a gradual reduction in its volume was observed in functional MRI studies [99]. An interesting observation, confirmed both in animal models and in studies involving healthy adults, concerns the reduction in the gray matter area of the hippocampus, which shows an inverse relationship with the level of IL-6 [100], which was also confirmed in studies on cancer patients [101]. Another possible mediating factor could be hormonal imbalance associated with hyperactivity of HPA axis [102]. Personality traits and temperamental factors might also be considered possible mediating factors. Allen (2017) presented possible associations between personality traits from the Big Five model of personality testing and depression. The aforementioned neurodevelopmental theory of depression highlights the role of personality traits. [103]. The interaction (Figure 1) seems to be very dynamic and involves biological as well as psychological factors.

## Figures and Tables

**Table 1:** Diagnostic criteria of depression according to ICD 11 and DSM 5 [14,15].

Major Depressive Disorder Diagnostic Criteria Single/Recurrent Episodes in DSM 5	Depressive Disorder in ICD 11
A. Five (or more) of the following symptoms have been present during the same 2-week period and represent a change from previous functioning; at least one of the symptoms is either (1) depressed mood or (2) loss of interest or pleasure. Note: Do not include symptoms that are clearly attributable to another medical condition. 1. Depressed mood most of the day, nearly every day, as indicated by either subjective report (e.g., feels sad, empty, hopeless) or observation made by others (e.g., appears tearful). (Note: In children and adolescents, can be irritable mood.) 2. Markedly diminished interest or pleasure in all, or almost all, activities most of the day, nearly every day (as indicated by either subjective account or observation). 3. Significant weight loss when not dieting or weight gain (e.g., a change of more than 5% of body weight in a month) or decrease or increase in appetite nearly every day. (Note: In children, consider failure to make expected weight gain.) 4. Insomnia or hypersomnia nearly every day. 5. Psychomotor agitation or retardation nearly every day (observable by others, not merely subjective feelings of restlessness or being slowed down). 6. Fatigue or loss of energy nearly every day. 7. Feelings of worthlessness or excessive or inappropriate guilt (which may be delusional) nearly every day (not merely self-reproach or guilt about being sick). 8. Diminished ability to think or concentrate, or indecisiveness, nearly every day (either by subjective account or as observed by others). 9. Recurrent thoughts of death (not just fear of dying), recurrent suicidal ideation without a specific plan or a suicide attempt or a specific plan for committing suicide. B. The symptoms cause clinically significant distress or impairment in social, occupational or other important areas of functioning. C. The episode is not attributable to the physiological effects of a substance or to another medical condition. Note: Criteria A–C represent a major depressive episode. Note: Responses to a significant loss (e.g., bereavement, financial ruin, losses from a natural disaster, a serious medical illness or disability) may include the feelings of intense sadness, rumination about the loss, insomnia, poor appetite and weight loss noted in Criterion A, which may resemble a depressive episode. Although such symptoms may be understandable or considered appropriate to the loss, the presence of a major depressive episode in addition to the normal response to a significant loss should also be carefully considered. This decision inevitably requires the exercise of clinical judgment based on the individual’s history and the cultural norms for the expression of distress in the context of loss. D. The occurrence of the major depressive episode is not better explained by schizoaffective disorder, schizophrenia, schizophreniform disorder, delusional disorder or other specified and unspecified schizophrenia spectrum and other psychotic disorders. E. There has never been a manic episode or a hypomanic episode. Note: This exclusion does not apply if all of the manic-like or hypomanic-like episodes are substance-induced or are attributable to the physiological effects of another medical condition.	Single episode depressive disorder is characterized by the presence or history of one depressive episode when there is no history of prior depressive episodes. A depressive episode is characterized by a period of depressed mood or diminished interest in activities occurring most of the day, nearly every day during a period lasting at least two weeks, accompanied by other symptoms such as difficulty concentrating, feelings of worthlessness or excessive or inappropriate guilt, hopelessness, recurrent thoughts of death or suicide, changes in appetite or sleep, psychomotor agitation or retardation and reduced energy or fatigue. There have never been any prior manic, hypomanic or mixed episodes, which would indicate the presence of a bipolar disorder. Recurrent depressive disorder is characterized by a history or at least two depressive episodes separated by at least several months without significant mood disturbance. A depressive episode is characterized by a period of depressed mood or diminished interest in activities occurring most of the day, nearly every day during a period lasting at least two weeks, accompanied by other symptoms such as difficulty concentrating, feelings of worthlessness or excessive or inappropriate guilt, hopelessness, recurrent thoughts of death or suicide, changes in appetite or sleep, psychomotor agitation or retardation and reduced energy or fatigue. There have never been any prior manic, hypomanic or mixed episodes, which would indicate the presence of a bipolar disorder.

**Table 2:** Examples of studies on IL-1 levels with different kinds of co-factors (X marks studies focused on mentioned area).

Study	Depression	Pharmacotherapy	Various Forms of Complementary and Alternative Treatment	Psychotherapy	Personality Traits, Individual Reaction to Stress
Zou W. et al. 2018 [33]	X	X			
Song C. et al. 2009 [34]	X	X	X		
Leo R. et al. 2006 [35]	X	X			
Hannestad J. et al. 2011 [37]	X	X			
Yu J.J. et al. 2015 [38]	X	X	X		
Rethorst C.D. et al. 2013 [39]	X		X		
Jazayeri S. et al. 2010 [40]	X	X	X		
Liu Y. et al. 2015 [42]	X	X	X		
Sun H. et al. 2010 [43]	X	X	X		
Cattaneo A. et al. 2016 [44]	X	X			
Rethorst C.D. et al. 2017 [45]	X	X	X	X	
Steptoe A. et al. 2007 [46]	X				X
Aschbacher K. et al. 2012 [47]	X				X
Spivak B. et al. 1997 [48]					X

**Table 3:** Examples of studies on IL-6 levels with different kinds of co-factors (X marks studies focused on mentioned area).

Study	Depression	Pharmacotherapy	Various Forms of Complementary and Alternative Treatment	Psychotherapy	Personality Traits, Individual Reaction to Stress
Sjogren E. et al. 2006 [49]					X
Lutgendorf S.K. et al. 1999 [50]	X				
Brydon L. et al. 2009 [51]					X
Krogh J. et al. 2014 [51]	X				
Pike J.L. et al. 2006 [53]	X				
Frommberger U.H. et al. 1997 [54]	X				
Leu S.J. 2001 (55)	X			X	
Trzonkowski P. et al. 2004 [56]	X				
Valkanova V. et al. 2013 [58]	X				
Hiles S.A. et al. 2012 [59]	X				
Duivis H.E. et al. 2013 [60]	X				
Basterzi A.D. et al. 2005 [62]	X	X			
Hasebe K. et al. 2017 [63]	X	X	X		
Taraz M. et al. 2013 [64]	X	X			
Pizzi C. et al. 2009 [65]	X	X			
Leo R. et al. 2006 [35]	X	X			
Bot M. et al. 2011 [66]	X	X			
Jazayeri S. et al. 2010 [40]	X	X	X		
Park M. et al. 2017 [68]	X	X			
Liu Y. et al. 2015 [42]	X	X	X		
Sun H. et al. 2012 [43]	X	X	X		
Leu S.J. et al. 2001 [55]	X		X		
Bull S.J. et al. 2009 [77]	X		X		
Abbasi S.H. et al. 2012 [69]	X	X	X		
Tolahunase M.R. et al. 2018 [70]	X		X		
Ranjbar E. et al. 2014 (71)	X	X	X		
Rethorst C.D. et al. 2013 [39]	X	X	X		
Oliver-Baxter J.M. et al. 2018 [72]	X		X		X
Rethorst C.D. et al. 2017 [45]	X		X		X
Euteneuer F. et al. 2017 [75]	X		X		
Benedetti F. et al. 2002 [74]	X		X		
Carney R.M. et al. 2016 [80]		X		X	
Lanquillon S. et al. 2000 [73]	X	X			
Maciukiewicz M. et al. 2015 [76]	X	X			
Moreira F.P. et al. 2015 [78]				X	
Thornton L.M. et al. 2009 [81]				X	

**Table 4:** Examples of research papers and meta-analyses of cognitive functioning among patients suffering from depression.

Cognitive Function Examples	Examples of Studies (Research and Meta-Analyses) of Those Functions among Depressed Patients
Executive functions: action planning, inhibition of action, change of the way of action	Krogh J. et al., 2014 [52] Świtalska J. 2013 [93] Talarowska M. et al. 2015 [89] McDermott Edmeier 2009 [95] Dunkin J.J. et al. 2000 [97] Rock P.L. et al. 2014 [88] Roca M. et al. 2015 [86]
Memory	Krogh J. et al., 2014 [52] Świtalska J. 2013 [93] Talarowska M. et al. 2015 [89] McDermott Edmeier 2009 [95] Dunkin J.J. et al. 2000 [97] Rock P.L. et al. 2014 [88] Roca M. et al. 2015 [86]
Attention	Świtalska J. 2013 [93] McDermott Edmeier 2009 [95] Dunkin J.J. et al. 2000 [97] Rock P.L. et al. 2014 [88] Roca M. et al. 2015 [86]
Verbal fluency	Krogh J. et al., 2014 [52] Talarowska M. et al. 2015 [89] Dunkin J.J. et al. 2000 [97] Rock P.L. et al. 2014 [88] Roca M. et al. 2015 [86]
Psychomotor speed, hand–eye coordination	Krogh J. et al., 2014 [52] Talarowska M. et al. 2015 [89] McDermott Edmeier 2009 [95] Dunkin J.J. et al. 2000 [97] Rock P.L. et al. 2014 [88] Roca M. et al. 2015 [86]

## Data Availability

https://www.ncbi.nlm.nih.gov/ and https://scholar.google.pl/, accessed on 12 December 2021.
